# Retrospective analysis of biochemical markers in COVID-19 intensive care unit patients

**DOI:** 10.1186/s43168-022-00129-7

**Published:** 2022-05-13

**Authors:** Sema Ketenci, İlkay Saraçoğlu, Recep Duranay, Çağrı Serdar Elgörmüş, A. Şükrü Aynacıoğlu

**Affiliations:** 1 Department of Medical Pharmacology, Faculty of Medicine, Istanbul Atlas University, Istanbul, Turkey; 2 Industrial Engineering, Faculty of Engineering, Istanbul Atlas University, 34408 Istanbul, Turkey; 3 Computer Engineering, Faculty of Engineering, Istanbul Atlas University, Istanbul, Turkey; 4 Emergency Medicine, Medicine Hospital, Istanbul Atlas University, Istanbul, Turkey

**Keywords:** COVID-19, Biomarkers, Intensive care unit, Biochemical parameters

## Abstract

**Background:**

The aim of the study was to evaluate the presence and effects of hematological and biological parameters in the diagnosis of the disease by performing blood tests on COVID-19 patients admitted to the intensive care unit (ICU).

**Results:**

Biochemical parameters from the blood samples of 279 patients who were confirmed to have COVID-19 and met the criteria for admission to the ICU were compared between discharged and deceased patients. Multiple logistic regression analysis was performed in terms of mortality and probability of being discharged. The predictive value of serum C-reactive protein (CRP), procalcitonin (PCT), lymphocyte, neutrophil, leucocyte, and platelet (PLT) levels was evaluated by measuring the area under the receiver operating characteristic curve (AUROC).

Comparisons made according to deceased and survival patients results revealed that while no statistically significant difference was observed between test groups lymphocyte and platelet-lymphocyte ratio values, statistically significant difference was found between the test groups regarding platelet, leukocyte, neutrophil, PCT, neutrophil-lymphocyte ratio (NLR), and thrombocyte count × neutrophil count/lymphocyte count (SII) values.

**Conclusions:**

This study showed that biochemical parameters examined are important in determining the prognosis of the disease and may be useful in determining the direction of the treatment process and predicting the risk of discharge or death after the initial evaluation of the patients in the ICU.

## Background

In December 2019, coronavirus disease 2019 (COVID-19) was reported in the Wuhan State of China caused by severe acute respiratory syndrome-coronavirus-2 (SARS-CoV-2) [1]. During the evolution of the disease, different clinical features and constantly changing laboratory parameters in its prognosis continue to make COVID-19 more deadly. Understanding the relationship between biological processes and clinical outcomes and expanding treatment options for diseases are of great importance, as COVID-19 infection is an epidemic situation that affects very large populations and causes mortality and morbidity. Early detection of inflammatory markers and rapid isolation of SARS-Cov-2 provide support for clinical advances and therapeutic approaches in disease management. Numerous clinical studies published on COVID-19 have focused on the evaluation of patient’s biochemistry and hemogram parameters [2–4]. In the course of the COVID-19 disease, biochemical markers play a critical role in the diagnosis, follow-up, and improvement of the treatment development process [5, 6].

In addition, the need for ICU follow-up in COVID-19 patients is planned according to the occupancy rate of the virus, and comorbid causes that will increase the mortality are also important issues [7]. It is thought that the results of the comparative analysis and the interpretation of the biochemical analyses of COVID-19 patients during their stay in the ICU will contribute to the prediction of the progression of the disease, treatment options, the timing of hospitalization, and predicting mortality.

Blood tests have an important role in early diagnosis of the disease, considering the information they provide to physicians regarding the inflammatory process. This information includes leukocyte count and characteristics such as neutrophil- or lymphocyte-dominance, C-reactive protein (CRP), organ damages, and the severity of the disease. Furthermore, biomarkers provide information regarding the nature of pneumonia, meaning that physicians can determine whether a disease is bacterial or due to other etiologies by analyzing blood test results [8]. Included in the complete blood counts (CBC) are values such as leucocytes, neutrophil-lymphocyte ratio (NLR), procalcitonin (PCT), platelet-lymphocyte ratio (PLR) and systemic inflammatory index (SII = thrombocyte count × neutrophil count/lymphocyte count), neutrophil and mean platelet (PLT) volume, and certain ratios of these values. Neutrophils are the most characteristic cell type among the leucocytes and are an important component of the immune system. Regulated by mast cells, epithelial cells, and macrophages, neutrophils also take part in inflammatory processes. The role of lymphocytes in both inflammation and infections is evident. While these parameters may be used as inflammatory markers by themselves, their ratios to one another may also be indicators of early inflammation [9–11]. Circulating leukocytes respond to stress by increasing neutrophils and reducing lymphocytes; the ratio of these two parameters is also used as an inflammatory marker [12].

The aim of the study was to evaluate the impact of the most appropriate hematological and biological parameters on the severity of COVID-19.

## Methods

### Study design

**Fig. 1:**
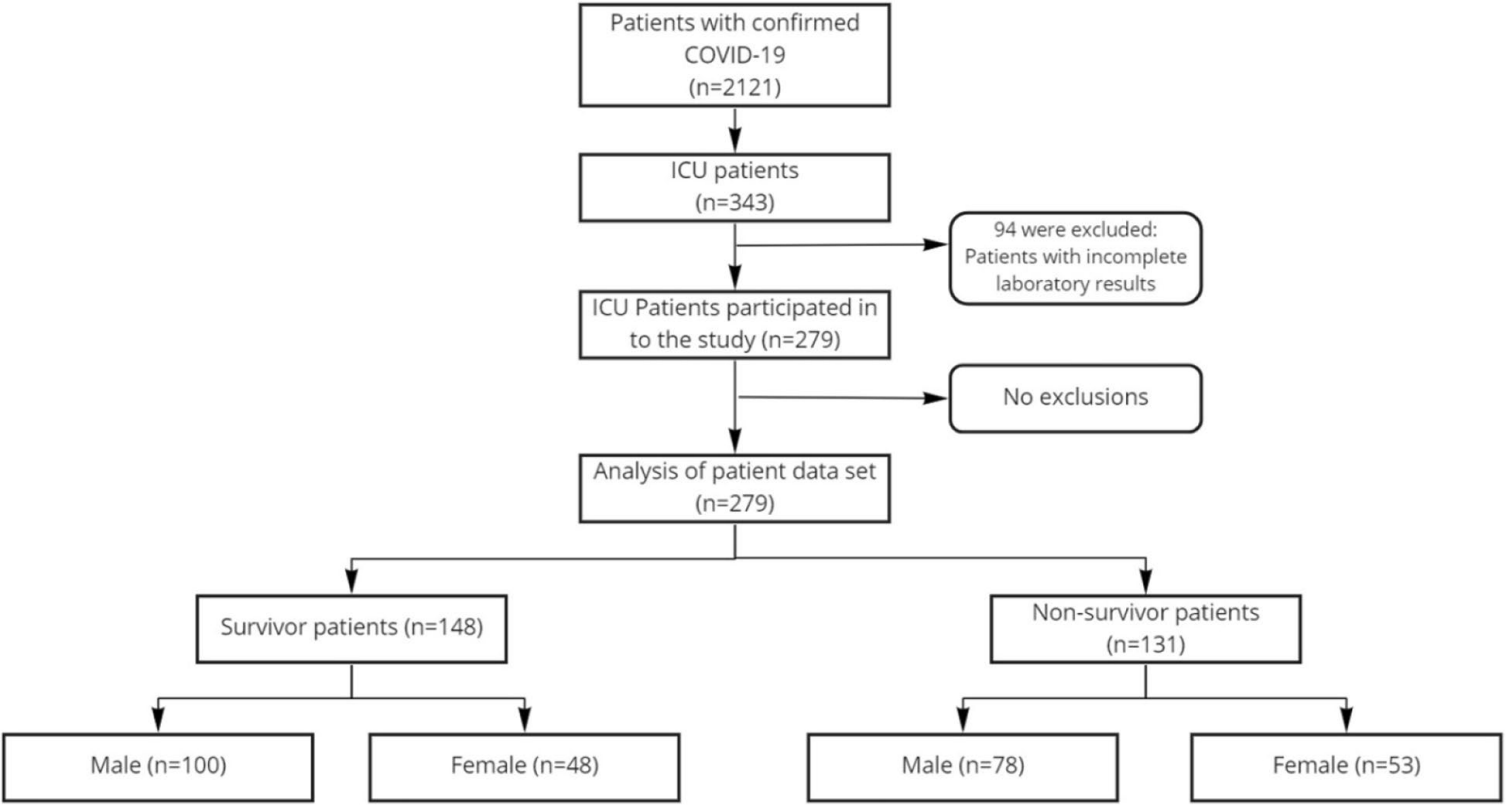
Patient flow of the study

Two-hundred seventy-nine COVID-19 patients (confirmed diagnosis with real-time RT-PCR assay), who were hospitalized at the Istanbul Atlas University Medicine Hospital between 6 October 2020 and 6 March 2021 and were consulted for assessment of the need for ICU admission, were collected using the hospital database (Fig. 1). Patient management for COVID-19 was planned according to the guidelines published and updated by the Turkish Ministry of Health [13, 14].

This retrospective study was carried out in line with research regulations, including the approval of the Istanbul Atlas University Local Ethics Committee dated 22 Sep 2021. This study also agrees with the principles of the Declaration of Helsinki of the World Medical Association.

### Data collection

Clinical and laboratory findings of these patients during consultation were obtained electronically and evaluated retrospectively. Patients data including age, gender, laboratory parameters obtained from blood samples, and the data obtained as a result of examination and recorded on the hospital database consultation form were analyzed. Respiratory rate ≥ 30 times/min, dyspnea and respiratory distress symptoms, arterial oxygen saturation/oxygen concentration ≤ 300 mmHg, bilateral infiltrates or multilobar involvement on chest computed tomography, thrombocytopenia, organ dysfunctions, the presence of multiple or particularly uncontrolled comorbidity, and troponin elevation are accepted as ICU admission criteria [13].

### Evaluation of data

All data were entered twice into a computer using Minitab® 19.2020.1 program fort to double-check to ensure accuracy. The counting data were expressed as multiple regression analyses for detection of dependent and independent variables and estimation of mortality. Binary logistic regression and area under the receiver operating characteristic curve (AUROC) analysis was performed by taking into account all neutrophil, lymphocyte, PLT, CRP, PCT, NLR, and SII variables, which appeared to have an effect. All statistical analyses were performed using IBM SPSS Statistics version 25.0, and findings were considered statistically significant *p* < 0.05.

## Results

A total 279 cases were included in this study, including 178 males and 101 females. Of 178 male patients, 78 (43.8%) died and 100 (56.2%) were discharged. Of 101 female patients, 53 (52.5%) died and 48 (47.5%) were discharged. The mean age of the female and male patients in the study was 67.15 ± 15.4 and 67.25 ± 15.3, respectively. Demographic characteristics of the patients are shown in Table 1. The mean age of the 279 patients who were discharged from the study was 62.5 ± 16.27 years, while the mean age of those who died was 72.4 ± 12.61 (*p* < 0.005). There were no statistically significant differences between deceased and discharged patients regarding the gender. Among the biochemical parameters, neutrophil, lymphocyte, platelet, CRP, PCT, NLR, and SII were found to be significantly different in the discharged and expired ICU patient groups (Table 2). However, there were no significant difference in leucocyte and PLR parameters between deceased and discharge groups.

**Table 1:** Demographic characteristics and hospitalization days in ICU patients (*n* = 279)

Parameters		Total	Deceased group	Discharged group	***p***-value
		**(*n*** **= 279)**	**(*n*** **= 131)**	**(*n*** **= 148)**	
**Gender**	**Female**	101 (36.2%)	53 (40.5%)	48 (32.4%)	0.164^(1)^
	**Male**	178 (63.8%)	78 (59.5%)	100 (67.6%)	
**Age (years)+**	**General**	67.222 ± 15.45	72.46 ± 12.61	62.59 ± 16.27	0.000*^(2)^
		69 (58–78)	75 (67–82)	64 (54.25–71)	
	**Female**	70.28 ± 15.9	73.91 ± 14.01	66.27 ± 17.01	
		75 (60–83)	77 (68–84.5)	64.5 (57.25–80.5)	
	**Male**	65.49 ± 14.96	71.47 ± 11.56	60.82 ± 15.68	
		67 (56.75–76)	71 (65.75–79.25)	63 (52–69)	
**Hospitalization days in ICU+**		11.6 ± 8.4	10.8 ± 7.1	12.2 ± 9.3	0.15^(2)^

+Mean ± standard deviation/median (interquartile range: Q1–Q3). *Significant *p*-value < 0.05. ^(1)^Chi-square test. ^(2)^2-sample *t*-test

**Table 2:** Laboratory tests of 279 ICU patients with COVID-19

Hematological Parameters	Deceased group (***n*** = 131)	Discharged group (***n*** = 148)	***p***-value
**Leucocyte (× 10**^**9**^**/L)**	14.7 ± 13.5	12.8 ± 6.7	0.198
**Neutrophil (× 10**^**9**^**/L)**	13.1 ± 13.3	10.8 ± 6.3	0.041*
**Lymphocyte (× 10**^**9**^**/L)**	0.9 ± 0.8	1.2 ± 1.2	0.007*
**Platelet (× 10**^**9**^**/L)**	242.2 ± 120.3	266.8 ± 106.7	0.018*
**CRP (mg/L)**	135.8 ± 86.8	104.5 ± 85.3	0.001*
**PCT (ng/ml)**	5.1 ± 31.8	2.5 ± 9.6	0.001*
**NLR (10**^**3**^ **cells/ μl)**	21.6 ± 19.7	15.7 ± 18.3	0.000*
**PLR (10**^**3**^ **cells/ μl)**	408.4 ± 314.6	369.7 ± 318.3	0.186
**SII (per μl)**	5280 ± 7837	4360 ± 5806	0.020*

+Mean ± standard deviation. *Significant *p*-value < 0.05. *ICU*, intensive care unit; *PCT*, Procalcitonin; *CRP*, C-reactive protein; *NLR*, neutrophil lymphocyte ratio; *PLR*, platelet lymphocyte ratio, *SII*, systemic inflamatory index. [Platelet × NLR (per μl)]. Mann-Whitney test was used

Interpretations are made with the odds, which are the ratio of the probability of being deceased to the probability of being discharged. According to the odds ratio values in Table 3, it was determined that the most important variable in the event of death was neutrophil and age. Age, neutrophil, CRP, NLR, and SII independent variables are observed to be independent risk variables on death due to COVID-19, with OR values of 1.0492, 1.0497, 1.0037, and 1.0365, respectively. Each unit increase of these variables increases the probability of mortality. A one-unit increase in age will increase the probability of an ex event 1.0492 times, provided other variables remain constant. Provided that other variables remain constant, a one-unit increase in neutrophil value will increase the probability of being an ex by 1.0497 times. According to the results in Table 3, it is seen that the SII variable reduces the mortality rate.

**Table 3:** Age, neutrophil, CRP, NLR, and SII, independent risk variables on death due to COVID-19, with OR

Odds ratios for continuous predictors			
	Odds ratio	95% ***CI***	***p***-value
**Age**	1.0492	(1.0277, 1.0710)	0.000
**Neutrophil**	1.0497	(1.0037, 1.0978)	0.034
**CRP**	1.0037	(1.0006, 1.0068)	0.021
**NLR**	1.0365	(1.0069, 1.0670)	0.015
**SII**	0.9998	(0.9997, 0.9999)	0.019

**Fig. 2:**
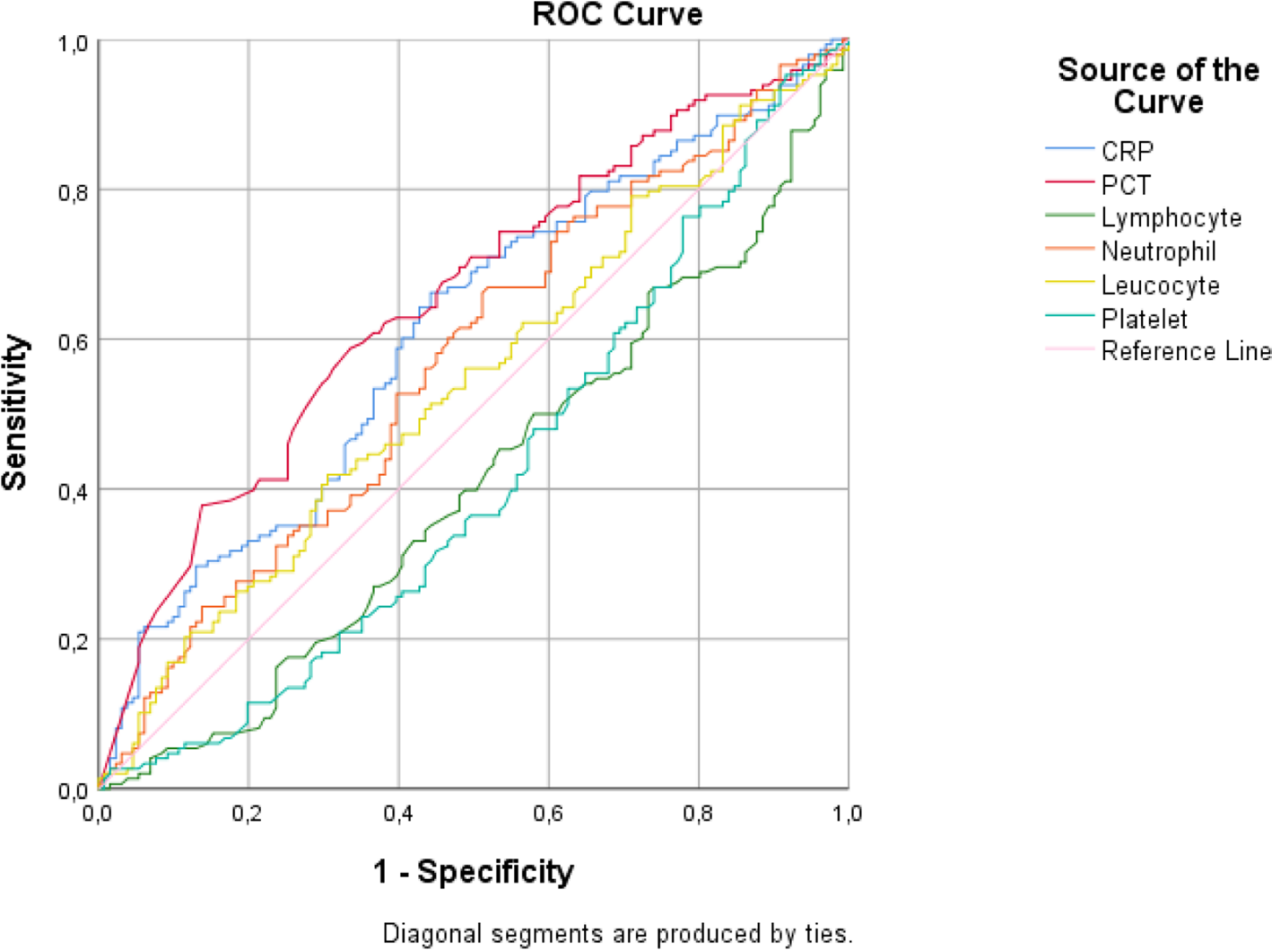
AUC of ROC curve of the CRP, PCT, lymphocyte, neutrophil, leucocyte and platelet values

The significance of CRP, PCT, lymphocyte, neutrophil, leucocyte, and platelet values according to ROC analysis was examined; CRP, PCT, lymphocyte, and platelet were found to be statistically significant. Our results showed that age indicator in the deceased group was significantly higher than that in the discharge group. The AUROC of the age predicting the deceased of ICU was the largest. The cutoff age point was 68.5; sensitivity and specificity were 68.7% and 63.9%, respectively. The second largest area of the AUC was NLR. NLR in the deceased group was significantly higher. The AUROC of the NLR predicting the deceased of ICU was the largest hematologic biomarker (Fig. 2, Table 4).

**Table 4:** The AUCROC of each independent risk or prediction factors

Indicators	AUC	Cutoff value	Sensitivity	Specificity	Lower and upper bound
**Age**	0.703	68.5	0.687	0.639	0.641–0.765
**Neutrophil**	0.437	10.21	0.455	0.455	0.365–0.508
**SII**	0.581	3078.1	0.595	0.595	0.514–0.648
**NLR**	0.649	9.2	0.606	0.610	0.584–0.714
**CRP**	0.613	106.1	0.597	0.593	0.545–0.677

## Discussion

In the fight against COVID-19 disease, the correct interpretation of biomarkers is very important in terms of diagnosing the severity of the disease, evaluating patient admission criteria, patient care, and standardization of treatment. In general, hematological markers such as CRP and PCT are accepted as important infection biomarkers of severe COVID-19 disease [15]. The rates of marker levels between severe and mild disease groups showed differences in most studies [16, 17]. The significantly higher PCT and CRP values in the ICU patients are related to the severe and adverse progress of COVID-19 [18–20]. Obtaining similar results in biochemical parameters in our study was consistent with other studies [21–24]. Their data showed that COVID-19 might mainly act on lymphocytes, particularly T lymphocytes. NLR was identified as an early risk factor for severe COVID-19, and most patients in the severe group showed higher levels of infection-related indicators such as CRP. In our study, as in other studies, NLR was shown to be important in determining the severity of COVID-19. High levels of NLR may be a strong prognostic indicator for worsening the severity of COVID-19 [25, 26]. Furthermore, Chan et al. in a meta-analysis concluded that NLR and PLR can be served as independent prognostic markers of disease severity in COVID-19 [27]. In our study, neutrophil, lymphocyte, platelet, NLR, PCT, SII, and CRP showed significant difference between survival and deceased groups. There are also some studies showing that T cells are important for the recognition and clearance of infected cells, particularly in the lungs of infected individuals. Thus, pathophysiology of COVID-19 is majorly associated with lymphocytes, especially T lymphocytes exaggerated inflammatory responses during the lung involvement [28, 29]. In this study, when the significance of CRP, PCT, lymphocyte, neutrophil, leucocyte, and platelet values according to ROC analysis was examined, CRP, PCT, lymphocyte, and platelet were found to be statistically significant (*p* < 0.05). Among the hematological parameters, the most prominent area of the AUCROC was for the CRP and PCT values. For the values here, the diagnostic test is easy to distinguish but difficult to interpret because the areas of the other values are close to the 0.5 line.

In addition, when the relationship of important biomarkers with COVID-19 prognosis in the intensive care stage is interpreted, estimates can be made with data on varying levels and regression analysis. These results can be very helpful in terms of ease of diagnosis and monitoring of response to treatment. SII was found to be significantly higher in deceased patients when compared with survival group, meaning that it can also be used as predictor of COVID-19 severity and outcome. COVID-19 ICU patients were more commonly observed in men than women (64% vs 36%) in our study [30, 31]. Although the age factor was found to be significantly different, the length of stay in the ICU patients did not have a significant effect on discharge or death. In correlation with other studies, no significant difference was found in the gender factor. The most obvious result in our cohort is that the probability of death of patients hospitalized in the ICU is directly related to age, neutrophils, CRP, NLR, and SII. Therefore, early diagnosis and promptly treatment initiation of critically ill individuals is issue of crucial importance.

As demonstrated by several studies [32–35], our results showed also the NLR in the survival group was significantly higher than non-survival group. Recently, a meta-analysis performed by Lagunas- Rengel [25] showed that the NLR values were found to increase in COVID-19 patients. Our study showed that CRP and neutrophil were significantly higher than that in the deceased group, and CRP and neutrophil were an independent risk factor for COVID-19 ICU patients. Therefore, the detection of CRP levels in ICU patients is of great value in assessing their condition. Our study also showed that SII was lower in the deceased group than survival group. According to univariate regression analysis, neutrophil, lymphocyte, platelet, CRP, NLR and SII biomarkers are variable coefficients that affect the probability of ex. In addition, according to binary logistic regression analysis, SII was found to be an independent protective factor for intensive care patients with COVID-19.

In conclusions, NLR, PCT, CRP, and SII can be considered as prognostic, predictive, or risk-stratifying factors of severe form of COVID-19.

### Study limitations

This study has several limitations. One of the limitation was the relatively small sample size and missing of several biochemical parameters. In addition, other variables including concomitant diseases and treatment protocols that are also important during the course of COVID-19 were not determined in this study.

## Abbreviations

ICU: Intensive care unit
PCT: Procalcitonin
CRP: C-reactive protein
NLR: Neutrophil lymphocyte ratio
PLR: Platelet lymphocyte ratio
SII: Systemic inflammatory index [platelet × NLR (per μl)]
